# lncRNA Profiles Enable Prognosis Prediction and Subtyping for Esophageal Squamous Cell Carcinoma

**DOI:** 10.3389/fcell.2021.656554

**Published:** 2021-05-28

**Authors:** Shujun Zhang, Juan Li, Huiru Gao, Yao Tong, Peilong Li, Yunshan Wang, Lutao Du, Chuanxin Wang

**Affiliations:** ^1^Department of Clinical Laboratory, The Second Hospital, Cheeloo College of Medicine, Shandong University, Jinan, China; ^2^Shandong Engineering & Technology Research Center for Tumor Marker Detection, Jinan, China; ^3^Shandong Provincial Clinical Medicine Research Center for Clinical Laboratory, Jinan, China

**Keywords:** ESCC, lncRNAs, prognosis, molecular subtyping, EMT

## Abstract

Long non-coding RNAs (lncRNAs) have emerged as useful prognostic markers in many tumors. In this study, we investigated the potential application of lncRNA markers for the prognostic prediction of esophageal squamous cell carcinoma (ESCC). We identified ESCC-associated lncRNAs by comparing ESCC tissues with normal tissues. Subsequently, Kaplan–Meier (KM) method in combination with the univariate Cox proportional hazards regression (UniCox) method was used to screen prognostic lncRNAs. By combining the differential and prognostic lncRNAs, we developed a prognostic model using cox stepwise regression analysis. The obtained prognostic prediction model could effectively predict the 3- and 5-year prognosis and survival of ESCC patients by time-dependent receiver operating characteristic (ROC) curves (area under curve = 0.87 and 0.89, respectively). Besides, a lncRNA-based classification of ESCC was generated using k-mean clustering method and we obtained two clusters of ESCC patients with association with race and Barrett’s esophagus (BE) (both *P* < 0.001). Finally, we found that lncRNA AC007128.1 was upregulated in both ESCC cells and tissues and associated with poor prognosis of ESCC patients. Furthermore, AC007128.1 could promote epithelial-mesenchymal transition (EMT) of ESCC cells by increasing the activation of MAPK/ERK and MAPK/p38 signaling pathways. Collectively, our findings indicated the potentials of lncRNA markers in the prognosis, molecular subtyping, and EMT of ESCC.

## Introduction

Esophageal cancer is one of the most common cancers worldwide, and it ranks seventh and sixth in terms of overall incidence and mortality, respectively (Bray et al., 2018). There are two histologic subtypes including esophageal squamous cell carcinoma (ESCC) and esophageal adenocarcinoma (AC) (Short et al., 2017). ESCC occupies over 90% of all esophageal cancer patients and the long-term outcome of ESCC is still limited with 5-year survival rate of 20% (Pennathur et al., 2013; Siegel et al., 2020). In general, prognostic factors may be helpful for better individual risk stratification and clinical outcome prediction. Traditional prognostic factors mainly depend on clinical and pathologic features, such as the history of drinking and smoking, tumor stage, and lymph node metastasis and so on, which have an effect on the overall survival (OS) in ESCC. However, the clinical course of ESCC patients with the same characteristics might be significantly different (Liu et al., 2012). Discovering new prognostic factors that improve the clinical outcome prediction of patients has been an urgent clinical need in ESCC.

As endogenous RNA transcripts with more than 200 nucleotides, long non-coding RNAs (lncRNAs) lack protein-coding potentials (Djebali et al., 2012; Iyer et al., 2015; Quinn and Chang, 2016). They are now known to act as scaffolds to modulate interactions between proteins and genes, as decoys to bind proteins, and as enhancers to regulate transcription of target genes (Huarte, 2015; Schmitt and Chang, 2016; Yue et al., 2016; Zhang et al., 2018; Blank-Giwojna et al., 2019). The discrepant expressions of lncRNAs, which have been demonstrated in various types of malignancies, possess significant regulatory effects on oncogenesis and tumor progression, indicating their potential oncogenic and tumor-suppressive roles (Hu et al., 2014; Li et al., 2018b; Wang et al., 2018). Growing evidence has suggested that unique lncRNA expression profiles can provide important prognostic information for patients with cancer (Cheng, 2018). Till today, several differentially expressed lncRNAs have been proved to be relevant for ESCC prognosis (Xie et al., 2014; Chen et al., 2015; Hu et al., 2015; Wang et al., 2015). However, previously published studies mainly focused on single lncRNAs as prognostic biomarkers. Little or nothing is known about the multi-lncRNA-based signature associated with OS in ESCC.

Our previous study has shown that the serumal and/or urinary exosome lncRNA profiles can be applied to the diagnosis and prognostic prediction of bladder cancer, colorectal cancer, breast cancer, and lung cancer (Li et al., 2017, 2018a; Xie et al., 2018; Zhan et al., 2018; Zhang et al., 2019). In the present study, we used publicly available The Cancer Genome Atlas (TCGA) RNA-seq expression data and evaluated the potential effectiveness of lncRNA biomarkers for prognosis and subtyping of ESCC. We constructed a prognostic model with selected lncRNA markers and further obtained a lncRNA-based subtype of ESCC by k-means method. Besides, we validated the biological role of lncRNA AC007128.1 by cellular assays in ESCC progression.

## Materials and Methods

### Study Design

In the present study, we were aimed at identifying lncRNA-based model for the prognostic prediction and subtyping of ESCC. Firstly, differential and prognostic lncRNA markers were identified from public TCGA ESCC RNA-seq datasets, and then the prognostic model was constructed by cox stepwise regression analysis. The model was evaluated with cross-validation and time-dependent receiver operating characteristic (ROC) methods and then compared with the clinical characteristics including stage, lymph node metastasis, and distant metastasis. With the lncRNA profiles, two lncRNA ESCC clusters were also constructed via the unsupervised clustering method, and the robustness of subtyping markers in two other datasets was verified. The potential function and mechanism of AC007128.1 were further investigated, which demonstrated high expression in ESCC cells and tissues and could promote cell migration and invasion by MAPK pathway.

### Patients Source

Tissue RNA-seq data were obtained from TCGA and Gene Expression Omnibus (GEO) (TCGA-ESCA and GSE53625). Complete clinical and histopathological information are available at TCGA and GEO websites as follows: https://tcga-data.nci.nih.gov/tcga/ and https://www.ncbi.nlm.nih.gov/geo/. The characteristics of ESCC patients are summarized in Supplementary Table 1.

### Unsupervised Discovery of lncRNA-Based Subtypes

The training dataset (*n* = 111) was used to discover ESCC subtypes. To narrow down markers and obtain meaningful clustering by lncRNA information, the list was firstly ranked by variance, and the top 1,000 lncRNAs with the larger variance were selected. Secondly, different clustering methods, including hierarchical clustering (HC) (single, complete, average, centroid, and ward) and non-hierarchical clustering (NHC), were compared. Finally, the k-means was chosen to cluster for ESCC. Differentially expressed lncRNA markers were obtained among the new clusters by using the limma package, and the top 50 differential lncRNAs were selected. These 50 marker sets were finally used as the signature to predict ESCC subtypes. Validation was performed on the two predefined validation datasets (*n* = 51 and 179).

### Cell Lines and Cell Culture

Three human ESCC cell lines (KYSE150, EC109, and TE-1) and two normal esophageal epithelial cell lines (Het-1A and HEEC) were bought from the Chinese Academy of Medical Sciences (CAMS), China. KYSE150 and TE-1 cells were cultured in Roswell Park Memorial Institute 1640 (RPMI-1640) medium supplemented with 10% fetal bovine serum (FBS) (Australia Origin, Gibco, Carlsbad, CA, United States) at 37°C in a humidified atmosphere containing 5% CO_2_. EC109, Het-1A, and HEEC cells were maintained in Dulbecco’s Modified Eagle’s Medium (DMEM) supplemented with 10% FBS at 37°C in a humidified atmosphere containing 5% CO_2_.

### Small Interfering RNAs, Short Hairpin RNAs, Plasmids and Lentivirus Transduction

Small interfering RNAs (siRNAs) or plasmids were transfected into ESCC cells using Lipofectamine 2000 (Invitrogen, United States) following the manufacturer’s instructions. The supernatant of the lentivirus-infected cells was substituted with a complete culture medium after 24 h. The stable cell lines were constructed using puromycin. PcDNA3.1-AC007128.1 and pcDNA3.1 empty vector (BoShang, Jinan, China) were extracted using the Endo-Free Plasmid Mini Kit (Omega Bio-Tek, United States). Sequences for short hairpin RNAs (shRNAs) and siRNAs are listed in Supplementary Table 2.

### Cell Viability Assay

Cell viability was examed using the Cell Counting Kit*-*8 (CCK*-*8) assay (Dojindo Labs). The optical density was assessed with a microplate reader (Bio-Rad, Hercules, CA, United States) at a wavelength of 450 nm.

### Transwell Invasion and Migration Assays

The transwell invasion assay was done using the transwell (Corning, NY, United States) and matrigel (BD Biosciences, San Jose, CA, United States). Briefly, inserts with 8-μm pores were coated with 8 μL of matrigel. Cells (∼7.5 × 10^4^) with 200 μL of serum-free medium were added into the upper compartment of the chamber. Subsequently, 600 μL of RPMI-1640 medium or DMEM containing 20% FBS was added into the lower chamber of the chamber as a chemo-attractant. After 36 h of incubation at 37°C in a humidified atmosphere containing 5% CO_2_, cells that migrated through the matrigel were fixed with methanol, stained with 0.1% crystal violet, and photographed using a microscope (Zeiss, Axio Observer). The cell number in five randomly selected fields from the central and peripheral regions of the filter was counted. The migration assay was conducted similarly without matrigel coating.

### Wound Healing Assay

Wound healing assay was conducted in six-well plates. When cells reached a confluence of 90–100%, a wound was made with a 200-μL pipette tip. Spreading cells between parallel lines were measured by an inverted microscope (Zeiss, Axio Observer) and photographed after 0 and 24 h.

### RNA Isolation and Real-Time PCR

Total RNA was obtained from cultured cells with RNA fast 2000 Reagent (Fastagen, Shanghai, China) and quantified with a NanoDrop spectrophotometer (Thermo Fisher Scientific, Waltham, MA, United States). Purified RNA was reversely transcribed into cDNA using oligo-dT and random primers with the PrimeScript^TM^ RT reagent Kit (TaKara, Dalian, China). Real-time PCR was performed on a CFX-96 real-time PCR System (Bio-Rad, Shanghai, China) using TB Green^TM^ Premix Ex Taq^TM^ (TaKara, Dalian China). Glyceraldehyde phosphate dehydrogenase (GAPDH) was employed as a housekeeping gene. The relative expressions of the target genes were calculated using the 2^–ΔΔCT^ method and subsequent log2 transformed. Primer sequences are displayed in Supplementary Table 3.

### Western Blotting Analysis

Total cell lysates were obtained by using the Western/IP lysis buffer (Beyotime, Haimen, China). Equal amounts of proteins were subjected to sodium dodecyl sulfate-polyacrylamide gel electrophoresis (SDS–PAGE) and then transferred onto polyvinylidene fluoride (PVDF) membranes (Millipore, United States). Membranes were immunoblotted with primary antibodies (Supplementary Table 4), followed by incubation with peroxidase-conjugated affinipure goat anti-mouse IgG or peroxidase-conjugated affinipure goat anti-rabbit IgG (Supplementary Table 4). Immunoreactive bands were visualized using the enhanced chemiluminescence system (Bio-Rad Laboratories). GAPDH was adopted as a loading control.

### RNA-seq Library Construction

For RNA-seq experiments with shCtrl and shAC007128.1 in KYSE150 cells, total RNA was isolated from cells with the RNeasy mini kit (Qiagen, Germany). Paired-end libraries were constructed using the TruSeq^TM^ RNA Sample Preparation Kit (Illumina, United States) according to TruSeq^TM^ RNA Sample Preparation Guide. Briefly, the mRNA containing poly-A were purified using poly-T oligo-attached magnetic beads.

After purification, the mRNA was cleaved into small fragments using divalent cations at 94°C for 8 min. The cleaved RNA fragments were amplified into first-strand cDNA using reverse transcriptase and random primers. Second-strand cDNA then was synthesized by DNA polymerase I and RNase H. Next, these cDNA fragments went through an end-repair process, the addition of a single “A” base, and then ligation of the adapters. The products were purified and enriched with PCR to create the final cDNA library. Purified libraries were quantified by Qubit^®^ 2.0 Fluorometer (Life Technologies, United States) and validated by Agilent 2100 bioanalyzer (Agilent Technologies, United States) to confirm the insert size and calculate the molar concentration. Clusters were generated by cBot with the library diluted to 10 pM and then sequenced on the Illumina NovaSeq 6000 (Illumina, United States).

The library was constructed and sequenced at Shanghai Sinomics Corporation.

### RNA Sequencing Analysis

Raw reads were preprocessed by filtering out rRNA reads, sequencing adapters, short-fragment reads, and other low-quality reads. Tophat v2.0.9 (Trapnell et al., 2009) was used to map the cleaned reads to the human hg38 reference genome with two mismatches. After mapping, Cufflinks v2.1.1 (Trapnell et al., 2012) was used to generate FPKM values for known gene models. Differentially expressed genes (DEGs) were identified using Cuffdiff (Trapnell et al., 2012). The *p*-value significance threshold in multiple tests was set by the false discovery rate (FDR) (Storey and Tibshirani, 2003). The fold changes (FCs) were also estimated according to the FPKM in each sample. The DEGs were selected using the filter criteria as follows: FDR ≤ 0.05 and FC ≥ 2.

### Statistical Analysis

A Cox regression model was applied to build the combined prognosis score (cp-score). Survival analysis was performed by KM curves and log-rank tests with a high-risk and low-risk group assignment relative to the median of cp-score. The time-dependent ROC curve was adopted to compare the performance of cp-score, TNM stage, lymph node metastasis, distant metastasis, and the combination of all factors. The effects of potential risk factors upon the survival time were assessed by multivariate Cox regression analysis. A *P*-value < 0.05 was considered statistically significant. Analysis for functional data were presented as mean ± SEM. The comparison between two groups was conducted by the Student’s *t*-test. All statistical analyses were performed using R software (version 3.6.0.), SPSS 17.0 (IBM, SPSS, Chicago, IL, United States) and GraphPad Prism 5.0 (GraphPad Software, LaJolla, CA, United States).

## Results

### Identification of Differential and Prognostic LncRNAs for ESCC

**FIGURE 1:**
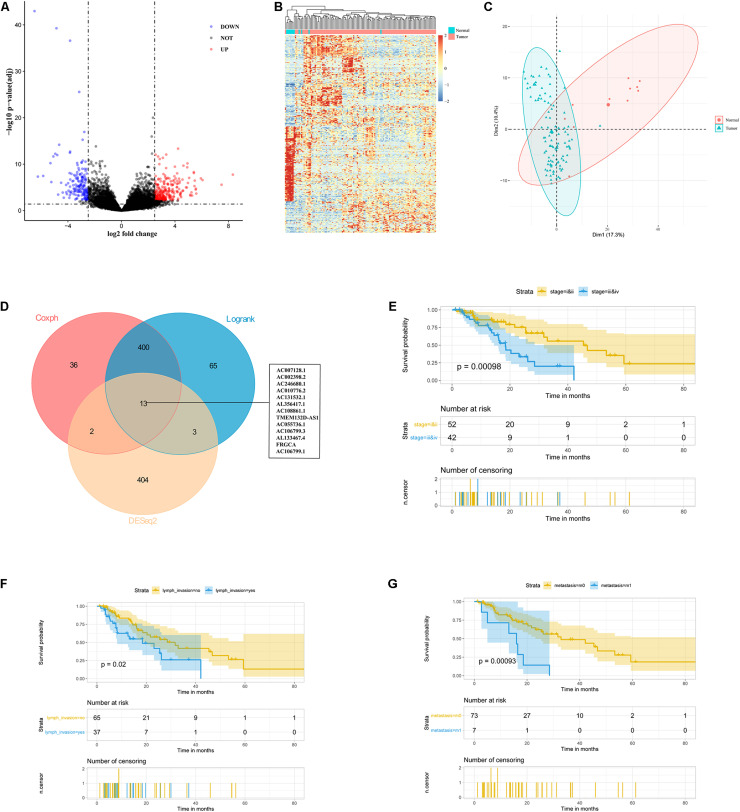
Identification of 13 lncRNAs as candidate prognostic markers for ESCC. **(A)** Volcano plot exhibited differentially expressed lncRNAs. **(B)** Heatmap analysis showed that ESCC patients could be distinguished by dysregulated lncRNAs. **(C)** PCA showed successful segregation between tumor and normal tissues. **(D)** The intersection of differentially expressed and prognostic lncRNAs by DEseq2, log-rank, and cox analysis. **(E–G)** Exploratory KM analysis (log-rank test) of the OS probability (with 95% confidence intervals) in ESCC patients [**(E)** TNM stage; **(F)** lymph node metastasis; and **(G)** distant metastasis].

Based on the RNA-seq data from TCGA database, we extracted lncRNA expression profiles and compared them between 111 ESCC tumor samples and 11 normal samples with DEseq2 package (Love et al., 2014), defined the best cutoff of 2.482 with the mean of absolute log2FC plus standard deviation of absolute log2FC, and found 422 lncRNAs with an FDR less than 0.05, including 257 up-regulated and 165 down-regulated lncRNAs (Figure 1A). The heatmap and principal component analysis (PCA) disclosed that ESCC patients and controls could be differentiated by the lncRNA expression profile (Figures 1B,C). These selected differential lncRNAs were considered as candidate prognostic markers for ESCC patients.

To select the prognostic lncRNAs, we analyzed 111 ESCC patients from TCGA with a follow-up of more than 1 month. The KM and univariate Cox proportional hazards regression (UniCox) methods were implemented to reduce the dimensionality. We obtained 413 overlapping lncRNAs based on the two algorithms, which were significantly correlated with the ESCC patients’ OS (Figure 1D). Based on the differential and prognostic lncRNAs, 13 lncRNAs were considered as candidate prognostic biomarkers for ESCC patients (Figure 1D).

Meanwhile, we examined the relationship between clinicopathological features and patients’ survival time. Patients with late-stage tumors or lymph node metastasis or distant metastasis had a shorter OS than those with early-stage tumors or without lymph node metastasis or distant metastasis (*P* = 0.00098, 0.02, and 0.00093 respectively, by log-rank test) (Figures 1E–G). However, gender, age, race, alcohol, Barrett’s esophagus (BE), and T stage were not correlated with the OS (Supplementary Figures 1A–E). Furthermore, UniCox showed the same results as the log-rank analysis (Table 1). Overall, these results suggested advanced stage, lymph node metastasis, and distant metastasis were related to poor prognosis of patients.

**TABLE 1:** Univariate and multivariable Cox regression analysis with covariates including cp-score, gender, age, race, alcohol, BE, stage, and T stage, lymph invasion and metastasis for overall survival.

Factor		Univariate coef					Multivariate coef			
		Exp (coef)	SE (coef)	*z*	*P*		Exp (coef)	SE (coef)	*z*	*P*
cp-score: L vs. H	−2.226	0.108	0.451	−4.936	**0.000**	−1.683	0.186	0.564	−2.986	**0.003**
Gender: M vs. F	0.369	1.446	0.525	0.702	0.483					
Age: >=60 vs. <60	−0.265	0.767	0.291	−0.91	0.363					
Race: White vs. Asian	0.322	1.38	0.74	0.435	0.663					
Alcohol_history: Yes vs. No	−0.281	0.755	0.295	−0.953	0.34					
BE: Yes vs. No	0.095	1.1	0.332	0.285	0.775					
Stage: III/IV vs. I/II	1.129	3.094	0.359	3.146	**0.002**	0.478	1.613	0.535	0.893	0.372
T stage: T3/4 vs. T1/2	0.254652	1.290013	0.328613	0.774931	0.43838					
Lymph invasion: Yes vs. No	0.718	2.05	0.314	2.288	**0.022**	−0.096	0.908	0.481	−0.2	0.842
Metastasis: M1 vs. M0	1.345	3.838	0.437	3.076	**0.002**	0.47	1.6	0.521	0.901	0.367

*Coef: The regression coefficients; Exp(coef): the exponentiated coefficients, also known as hazard ratios. SE(coef): The standard error of hazard ratios; BE: Barrett’s esophagus. The significant differences (P-value < 0.05) were highlighted with the bold values.*

### LncRNA-Based Prognostic Prediction Model for ESCC

**FIGURE 2:**
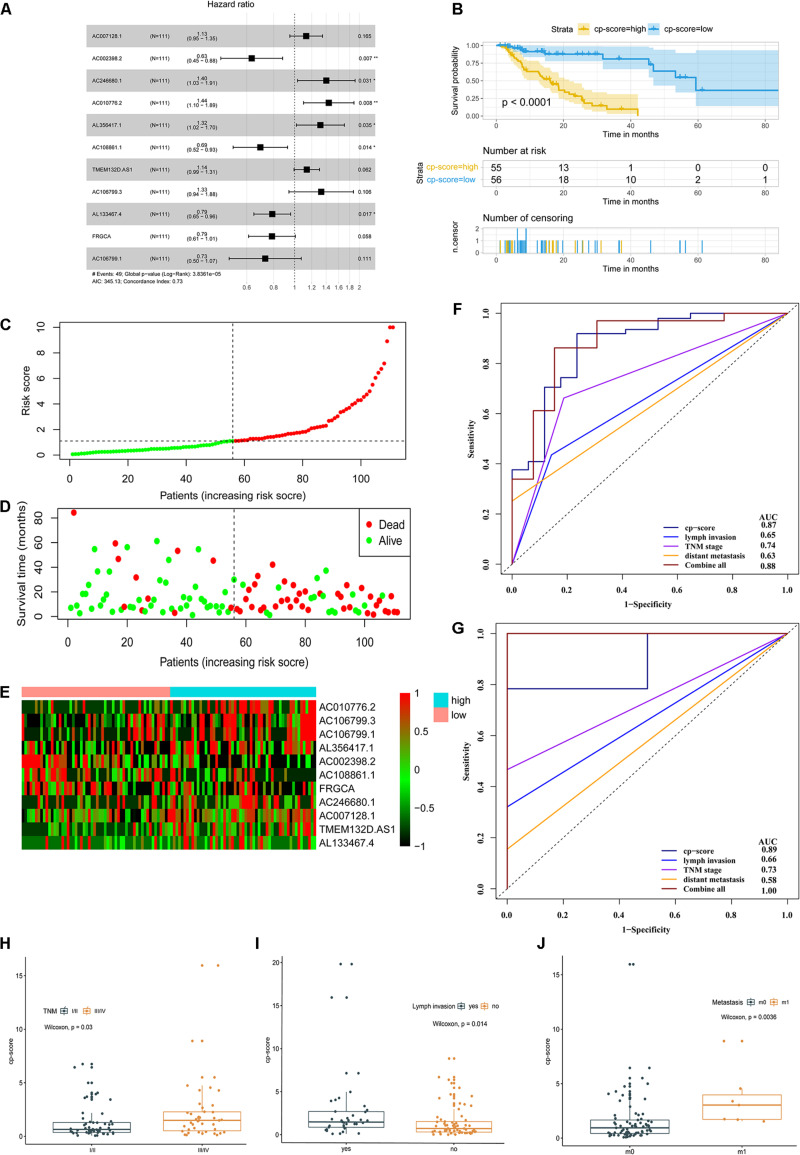
A prognostic model of ESCC survival based on 11 differential and prognostic lncRNAs. **(A)** Forest plot illustrated the HR and 95% CI of the 11 lncRNAs by the multivariate Cox regression. HR, hazard ratio; CI, confidence interval. **(B)** OS curves of ESCC patients with the low and high risk groups according to the cp-score in TCGA cohort. **(C–E)** The distribution of 11-lncRNA risk score **(C)**, OS status **(D)**, and corresponding lncRNA expression profiles **(E)**. The intersection of horizontal and vertical dotted lines represents the risk score of the person ranked in the middle. **(F)** Time-dependent ROC and corresponding AUCs for 3-year survival predicted by cp-score, TNM stage, lymph node metastasis, distant metastasis, and the combination of all these factors. **(G)** ROC and corresponding AUCs for 5-year OS predicted by cp-score, TNM stage, lymph node metastasis, distant metastasis, and the combination of all these factors. **(H)** LncRNA analysis of cp-score of patients with stage I/II and stage III/IV disease. **(I)** cp-score in patients with and without lymph node metastasis. **(J)** cp-score in patients with and without distant metastasis.

To construct the prognostic model, the 13 candidate lncRNAs were subjected to cox stepwise regression analysis, and 11 out of 13 lncRNAs were selected to build an optimum prognostic model, with a c-index of 0.73 indicating good discrimination (Figure 2A). Supplementary Table 5 lists the characteristics of 11 prognostic lncRNAs including permutation *P*-values, hazard ratios, and coefficients and so on. Figure 4E and Supplementary Figures 2A–J illustrate the KM curves for these lncRNAs. We introduced a cp-score for the prognostic prediction of ESCC according to Luo et al. (2020). The cp-score was obtained according to the coefficients from cox regression and separated the patients into high-risk and low-risk groups. KM curves were performed by using the dichotomized cp-score. The result showed that the low-risk group had significantly better survival time compared with the high-risk group (*P* < 0.0001) (Figure 2B). Figures 2C–E show the distribution of the risk score, OS status and the corresponding expression profiles of 11 lncRNAs, which were ranked according to the cp-score value. The mortality of high-risk patients was higher than low-risk patients.

Furthermore, we used a time-dependent ROC curve (Heagerty et al., 2000) to characterize the prognostic potential of the cp-score, pathological stage, lymph node metastasis, distant metastasis, and the combination of all above factors. The prognostic prediction model could effectively predict the 3- and 5-year prognosis and survival of ESCC patients by time-dependent ROC curves with area under curve 0.87 and 0.89, respectively (Figures 2F–G). Compared with pathological stage, lymph node metastasis, and distant metastasis, the cp-score both showed a better ability to predict prognosis in the 3- and 5-year (Figures 2F–G). Besides, the combination of cp-score and clinicopathologic characteristics significantly improved the performance to predict the 5-year prognosis [AUC-ROC:1.00] (Figure 2G) compared with the cp-score or clinicopathologic features alone, including TNM stage, lymph node metastasis, and distant metastasis.

Next, we assessed the association between cp-score and the TNM stage, T stage, the presence of lymph node metastasis, and distant metastasis of ESCC. Patients with stage I/II disease had lower cp-scores than those with stage III/IV disease (*P* = 0.03; Figure 2H). Similarly, the cp-scores of patients with lymph node and distant metastasis were significantly higher compared with those without lymph node and distant metastasis (*P* = 0.014 and 0.0036; Figures 2I,J). The cp-scores of patients with T3/4 tumors were moderately higher compared with those with T1/2 tumors (*P* = 0.18; Supplementary Figure 2K). These results demonstrated that the cp-score was correlated with the tumor stage and might be used to predict the TNM stage, lymph node, and distant metastasis. Multivariate Cox regression analysis indicated that the cp-score was an independent prognostic factor for ESCC (Table 1).

### lncRNA-Based Subtyping of ESCC

**FIGURE 3:**
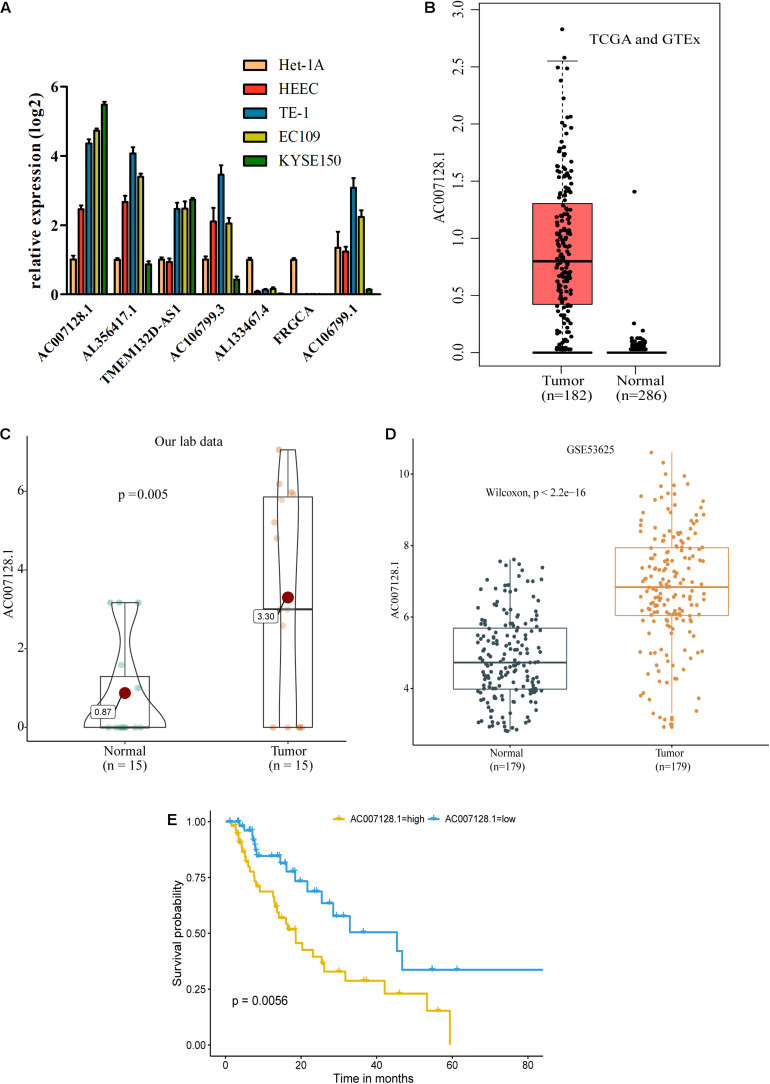
AC007128.1 is up-regulated and linked to poor prognosis in ESCC. **(A)** Comparison of seven lncRNA expressions in ESCC and normal cell lines. Het-1A and HEEC represent normal cell lines, and others represent tumor cell lines. **(B–D)** Relative expression of AC007128.1 in ESCC tissues was analyzed by using GEPIA: http://gepia.cancer-pku.cn **(B)**, our lab **(C)**, and GEO datasets **(D)**. **(E)** KM survival analysis of ESCC patients with high or low AC007128.1 expression (defined by the median) in TCGA. Statistical analysis was performed using the two-sided log-rank test.

We used unsupervised clustering to generate lncRNA-based subtypes of ESCC. Figure 3A illustrates the schematic diagram for sample clustering. Firstly, we compared different clustering methods, including HC (single, complete, average, centroid, and ward) and NHC methods (*k*-means). Ward showed better results among HC methods (Supplementary Figures 3A–E). The quality of the single clustering solutions could be assessed with average silhouette width as a measure for clustering quality, so we compare the clustering ability between ward and *k*-means methods by silhouette plot. The results showed ward method had a lower silhouette width (0.17) than *k*-means (0.2), which indicated that the clustering quality of ward method was inferior to that of *k*-means (Supplementary Figures 3G,H). We finally chose the k-means to cluster for ESCC. Using the 111 ESCC patients as a training dataset, we obtained two clusters with 50 lncRNAs that were different expression between the two clusters and clinical data were indicated by the annotation bars above the heatmap (Figure 3B). Silhouette analysis of the clusters showed a good clustering quality with average width 0.54 (Figure 3C). We also validated the clusters from residual 57 TCGA ESCC tumor samples with a follow-up of less than 1 month, which showed a strong positive silhouette width (average = 0.61) (Figures 3D,E). Furthermore, we also observed the robustness of 50 lncRNAs in another independent dataset from GEO (GSE53625) (Figures 3F,G). Taken together, these results indicated that k-means fitted the respective cluster for ESCC. Among these lncRNAs, four were also found in the differential gene list, while none were found in the prognostic marker list (Supplementary Figure 3I).

To explore the clinical significance of the two subtypes, we systematically tested the associations between the subtype and clinical characteristics, including TNM stage, distant metastasis, lymph invasion, grade, BE, location, tobacco, alcohol, age, sex, race, and survival outcomes (Supplementary Table 6, Figures 3H,I, and Supplementary Figures 3J,K). There was no survival difference between cluster 1 and cluster 2 in all three datasets (Supplementary Figures 3J,K, both *P* > 0.05, log-rank test). Cluster 1 were frequently observed to correlate with the white race and those with BE which was considered as a precancerous lesion (Figures 3H,I, both *P* < 0.05, chi-square test). These differences in race and BE with unsupervised lncRNA signatures might implicate the discrepancy of the intrinsic biological processes in each cluster group.

### AC007128.1 Is Highly Expressed in ESCC With Poor Prognosis

**FIGURE 4:**
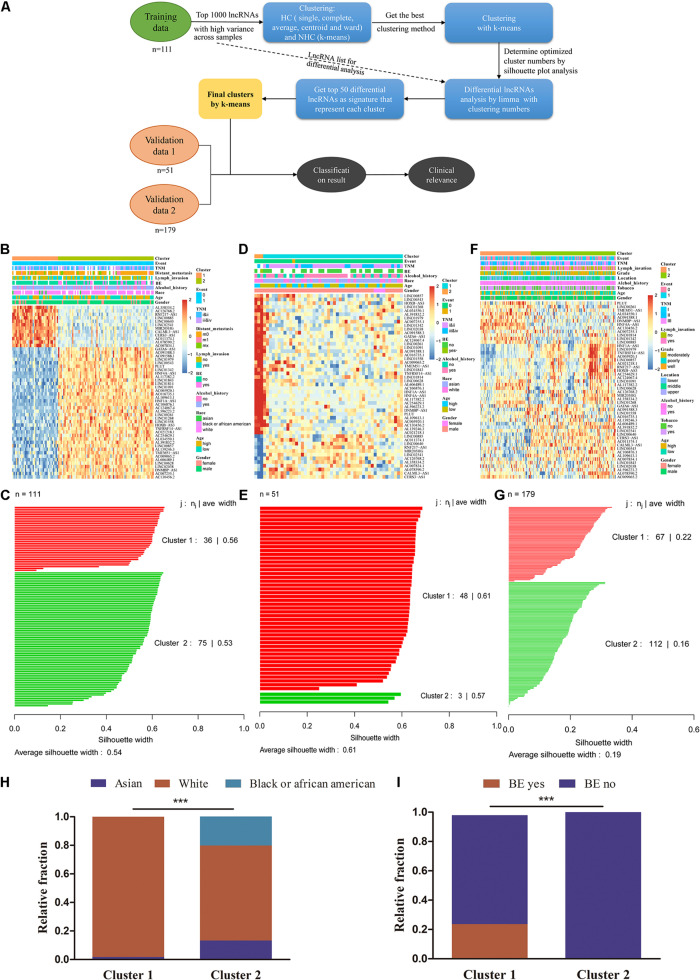
lncRNA subtyping analysis in 341 ESCC patients. **(A)** Schematic diagram for ESCC sample clustering. **(B)** *k*-means clustering of lncRNA markers identified two subtypes in the training cohort. The annotation bars above the heatmap indicate the clinical and molecular features. Patients without related information were colored in white. **(C)** Silhouette analysis of the clusters in the training data. **(D,E)** Subtypes of validation using the 50 markers in other 51 TCGA ESCC samples. **(F,G)** Predicted subtypes/clusters of validation using the 50 markers in 179 independent GEO ESCC samples (GSE53625). **(H)** The proportion of patients with Asian to White or Black ESCC in two clusters (chi-square test, ***P < 0.001). **(I)** The proportion of patients with and without BE in two clusters (chi-square test, ***P < 0.001). BE, Barrett’s esophagus.

To explore possible connections between lncRNAs and carcinogenesis and ESCC development, we evaluated the expressions of seven out of 11 prognostic lncRNA markers in ESCC cell lines and normal esophageal epithelial cell lines by RT-qPCR because the expressions of the other four lncRNAs were too low to detect. Of the seven lncRNAs, we found that AC007128.1 had a higher expression in three established ESCC cell lines (KYSE150, TE-1, and EC109) compared with normal cell lines (Het-1A and HEEC), and the expression of AC007128.1 was the highest among the seven lncRNAs (Figure 4A). We also detected the level of AC007128.1 in the nuclear and cytoplasmic fractions of KYSE150 and EC109 cells and found that AC007128.1 is mainly enriched in the nucleus (Supplementary Figures 4A,B). We also found a higher expression of AC007128.1 in human ESCC samples from three independent datasets (Figures 4B–D). Meanwhile, increased AC007128.1 expression was related with a shorter OS in TCGA ESCC samples (Figure 4E), which was consistent with a previous study (Liu et al., 2019). These data demonstrated that the expression of AC007128.1 was upregulated in both ESCC tissues and cells, and associated with poor prognosis in ESCC. Therefore, we focused on AC007128.1 for further research.

We further analyzed the correlation between the AC007128.1 expression and the clinicopathologic characteristics of the ESCC patients. The expression of AC007128.1 was associated with the TNM stage (*P* = 0.039), gender (*P* = 0.01), and age (*P* = 0.046) in TCGA dataset (Supplementary Table 7). The AC007128.1 expression was associated with grade (*P* = 0.013) in GSE53625 (Supplementary Table 8). There was no statistical association between the AC007128.1 expression and other clinical characteristics in the two datasets. Based on these data, we postulated a role for AC007128.1 in ESCC pathology.

### Identification of AC007128.1 as a Regulator of ESCC Cell Migration and Invasion

**FIGURE 5:**
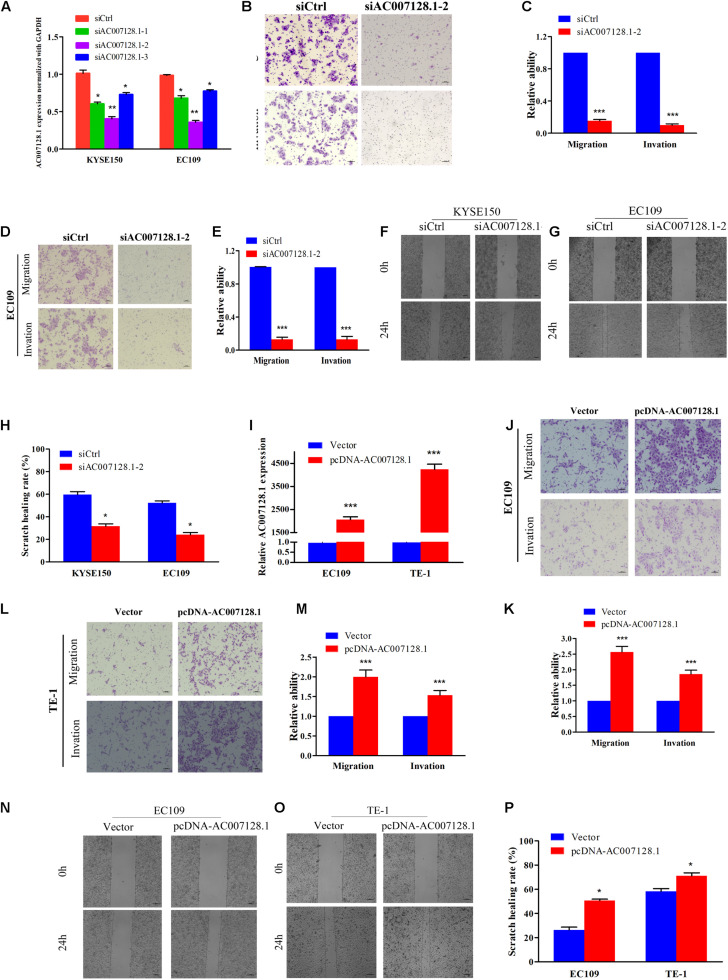
Effects of AC007128.1 expression on ESCC cell aggressiveness. **(A,I)** qPCR analysis of AC007128.1 expression in si-AC007128.1-1, si-AC007128.1-2, si-AC007128.1-3, and pcDNA-AC007128.1-treated ESCC cells. **(B–E)** Representative micrographs of the transwell assay showing the migration and invasiveness of AC007128.1-depleted cells. **(F–H)** Representative micrographs of the wound healing assay showing the motilities of AC007128.1-depleted cells. **(J–M)** Representative micrographs of the transwell assay showing the migration and invasiveness of AC007128.1-overexpressing cells. **(N–P)** Representative micrographs of the wound healing assay showing the motilities of AC007128.1-overexpressing cells. Data represent mean ± SEM from three independent experiments. *P < 0.05, **P < 0.01, and ***P < 0.001 by Student’s *t*-test as compared with the corresponding control.

To explore the biological effects of AC007128.1 in the tumorigenesis and development of ESCC, three specific siRNAs targeted to AC007128.1 were used to reduce its expression in two ESCC cell lines, KYSE150 and EC109. We showed that si-AC007128.1-2 exhibited a better silencing efficiency (Figure 5A). CCK-8 assay revealed that depletion of AC007128.1 did not affect the proliferation of KYSE150 and EC109 cells (*P* > 0.05; Supplementary Figures 4C,D). Next, we assessed the effect of AC007128.1 depletion on cell migration and invasion in ESCC. We found that AC007128.1 depletion dramatically inhibited cell migration, invasion, and wound closure in both KYSE150 and EC109 cell lines (Figures 5B–H). We also observed the same inhibitory effects on the migration and invasion in TE-1 cells (Supplementary Figures 4E–G). Transwell assay (Figures 5I–M) and wound healing assay (Figures 5N–P) indicated that over-expression of AC007128.1 in EC109 and TE-1 cells dramatically promoted cell migration and invasion. However, it had no significant effect on cell growth (Supplementary Figures 4H,I). Collectively, these results suggested that AC007128.1 promoted migration and invasion in ESCC cell lines.

**FIGURE 6:**
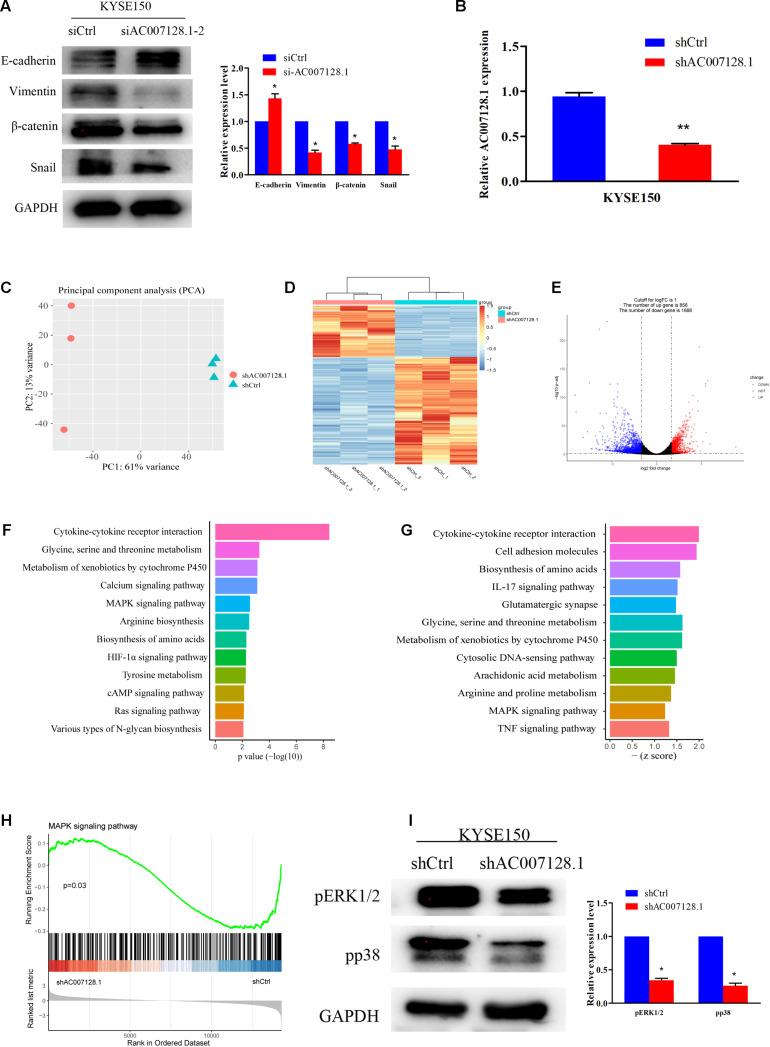
AC007128.1 activates MAPK/ERK and MAPK/p38 signaling pathways. **(A)** AC007128.1 depletion resulted in the increased expression of epithelial marker and decreased expressions of mesenchymal markers. **(B)** qPCR analysis of AC007128.1 expression in shAC007128.1- and shCtrl-treated ESCC cells. **(C)** PCA showed successful segregation between shAC007128.1 and shNC groups. **(D)** Heatmap analysis showed that shAC007128.1 cells could be distinguished by dysregulated lncRNAs. **(E)** Volcano plot exhibited differentially expressed lncRNAs. **(F)** KEGG analysis indicated the down-regulated signaling pathway by repressed genes in AC007128.1-depleted cells. **(G)** GSEA showed the repressed signaling pathway in AC007128.1-depleted cells. **(H)** GSEA indicated that AC007128.1 depletion might inhibit the MAPK signaling pathway. **(I)** Depletion of AC007128.1 obviously decreased the expressions of p-ERK1/2 and p-p38. *P < 0.05 and **P < 0.01 by Student’s t-test as compared with the corresponding control.

Epithelial-mesenchymal transition (EMT) plays an important role in tumorous migration and invasion (Yang and Weinberg, 2008; De Craene and Berx, 2013; Lamouille et al., 2014; Pang et al., 2018; Dongre and Weinberg, 2019). We further detected the expressions of EMT markers, including the epithelial marker E-cadherin and the mesenchymal markers, Vimentin, β-catenin, and Snail in AC007128.1-depleted cells. Figure 6A show that depletion of AC007128.1 resulted in a significantly increased expression of E-cadherin at the protein level, while it led to dramatically decreased expressions of Vimentin, β-catenin, and Snail at the protein level. These results indicated that AC007128.1 might promote EMT, thus affecting cell migration and invasion in ESCC.

### AC007128.1 Activated MAPK/ERK and MAPK/p38 Signaling Pathways

To explore the molecular mechanism, through which AC007128.1 promoted migration and invasion of ESCC, we constructed an shRNA specific to human AC007128.1, which was used to successfully reduce the expression of AC007128.1 at the mRNA level (Figure 6B), and further performed RNA-sequencing in KYSE150 cells. Global reprogramming of the ESCC transcriptome was detected in AC007128.1-depleted cells (Figure 6C), in which more than 2,500 genes were differentially expressed compared with the shCtrl group (Figure 6D), including 856 upregulated genes and 1,688 downregulated genes (Figure 6E). The Kyoto Encyclopedia of Genes and Genomes (KEGG) pathway enrichment analysis of the repressed genes showed that these down-regulated genes were mainly enriched in the following pathways including mitogen-activated protein kinase MAPK signaling pathway, cAMP signaling pathway, HIF-1α signaling pathway, Ras signaling pathway, and cytokine-cytokine receptor interaction, which were known to be important in cancer (Figure 6F). Moreover, gene set enrichment analysis (GSEA) for all genes revealed that some signaling pathways were depressed, such as MAPK signaling pathway, cell adhesion molecules, IL-17 signaling pathway, TNF signaling pathway, and cytokine-cytokine receptor interaction (Figure 6G). The results of KEGG and GSEA indicated that AC007128.1 might contribute to EMT via these signaling pathways in ESCC.

MAPK signaling pathway was identified as inhibited pathway in both KEGG and GSEA analysis when AC007128.1 was depleted in KYSE150 cells (Figures 6F–H). Previous studies has been implicated that the MAPK signaling pathway take part in regulating EMT of different cancers, including ESCC (Huang et al., 2004; Hu et al., 2017; Hawsawi et al., 2018; Chen et al., 2019; Peng et al., 2019), Therefore, we hypothesized that AC007128.1 might affect cell EMT by deregulating the MAPK signaling pathway. To test this hypothesis, we explored the effect of AC007128.1 on the activity of MAPK signaling. As expected, we found that depletion of AC007128.1 decreased the expressions of phosphorylated ERK1/2 (p-ERK1/2) and phosphorylated p38 (p-p38) in KYSE150 (Figure 6I). It has been shown that ERK1/2 (Wong et al., 2012; Wang et al., 2020; Zhang F. et al., 2020) and p38 (Wang et al., 2012; Ma et al., 2016) pathways are critical in EMT regulation for ESCC. Therefore, we inferred from the above-mentioned results that AC007128.1 affected cell EMT via deregulating MAPK signaling pathway.

## Discussion

In recent years, more and more novel lncRNAs have been identified and the roles of lncRNAs in cancer development have been increasingly studied (Yang et al., 2014; Bhan et al., 2017; Peng et al., 2017). In our previous study, we have shown the effectiveness of lncRNA signatures in diagnosis, prognostic prediction, and drug-resistance in four common cancers, namely breast cancer, colorectal cancer, bladder cancer, and lung cancer (Li et al., 2017, 2018a; Xie et al., 2018; Zhan et al., 2018; Zhang et al., 2019). Here, we identified an 11-lncRNA model that was associated with tumor OS in ESCC patients, constructed a lncRNA-based molecular subtype of ESCC, and found that lncRNA AC007128.1 might mediate ESCC EMT through mitogen-activated protein kinases/extracellular signal-regulated kinase (MAPK/ERK) and MAPK/p38 signaling pathways.

In the present study, we developed a prognostic prediction model using 11 selected lncRNA markers from TCGA ESCC RNA-seq dataset and found that this model could effectively distinguish ESCC patients with different prognoses. Moreover, multivariable analysis confirmed that this model was considered as an independent prognostic risk factor. The discrimination potential of the cp-score was superior to other prognostic risk factors (TNM stage, lymph node metastasis, and distant metastasis). We also compared the prognostic ability of this model with those of having been reported prognostic biomarkers for ESCC. The results demonstrated that the 11-lncRNA signature had a higher fidelity than other signatures (Li et al., 2014; Mao et al., 2018; Zhang L. et al., 2020). The combination of cp-score and clinical characteristics improved the 5-year survival prediction, which could identify patients needing more aggressive treatment and surveillance. We also found that the cp-score was correlated with the staging, as well as the lymphatic and distant metastases of ESCC. These results suggested that this model was also useful for detecting staging, lymphatic, and distant metastasis of ESCC. Although the 11-lncRNA model showed potential prognostic biomarkers for esophageal carcinoma, one limitation should be taken into consideration: the present study is short of other independent datasets to show its fidelity as prognostic biomarkers for esophageal carcinoma, and the distribution of clinical features in TCGA dataset might be distinct from those of other datasets, making it unsuitable for other subjects. Therefore, our results should be further validated in other clinical samples in the future.

Molecular subtyping is considered as a favorable source of disease stratification (Guinney et al., 2015; Sjodahl et al., 2017), while similar lncRNA-based subtyping is still lacking. With a k-means clustering analysis (Kakushadze and Yu, 2017), we could divide 111 ESCC patients from TCGA into two molecular subgroups based on 50 lncRNA markers, and its performance was again confirmed in internal and external datasets. The lncRNA-based subtyping showed good clustering capability and could be treated as a potential tool for ESCC molecular subtyping. We further studied the correlation between the lncRNA-based subtyping and clinical factors, including TNM stage, distant metastasis, lymph invasion, grade, BE, tumor site, alcohol, tobacco, age, sex, race, and survival outcomes. We found a relation between lncRNA-based clusters and race, alcohol, BE, and TNM stage in TCGA dataset, which might imply that these clinical variables partially affected the different transcriptions of lncRNAs. However, the study on subtyping was limited by clinical samples that all samples were collected from available public data. Further verification with multi-center clinical samples is still necessary to adequately assess the performance of the lncRNA-based subtype.

We presumed that lncRNA markers in the prognostic model might play important roles in the carcinogenesis and development of ESCC. Therefore, elucidating the underlying mechanism might provide potentially therapeutic targets of ESCC. In this study, we found lncRNA AC007128.1, which was significantly up-regulated in ESCC tissues from three independent datasets, and correlated with OS of ESCC in TCGA, However, there is one limitation to the validation of lncRNA expression: we didn’t detect the expression level of AC007128.1 by RT-qPCR or other method in new ESCC tissue samples due to limited corresponding samples. In view of this limitation, we attempted to prove the reliability of AC007128.1 by detecting the expression in ESCC cell lines and performing functional experiment *in vitro*. The results showed that AC007128.1 was upregulated in ESCC cell lines compared with normal esophageal epithelial cell lines and its depletion could suppress migration and invasion of ESCC cells *in vitro*. EMT is an important biological event during human cancer progression (De Craene and Berx, 2013; Pastushenko et al., 2018; Pastushenko and Blanpain, 2019). Reduced AC007128.1 could increase the expressions of epithelial markers and decreased the expressions of mesenchymal markers. Taken together, these results indicated that AC007128.1 might be closely related to cancer progression and play a tumor progressive role in ESCC.

It is urgently necessary to elucidate the molecular mechanisms underlying ESCC progression. We found that the deletion of AC007128.1 decreased the activation of MAPK/ERK and MAPK/p38 signaling pathways, but the exact mechanism by which AC007128.1 acts on the MAPK signaling pathway requires further study. Several published studies have also shown that the MAPK signaling pathway is activated in the process of tumorigenesis, metastasis, and angiogenesis of multiple human malignancies, including ESCC (Burotto et al., 2014; Hu et al., 2017). We speculated that AC007128.1 might promote migration and invasion of ESCC by increasing the activation of MAPK/ERK and MAPK/p38 signaling pathways.

Collectively, we identified lncRNA markers for prognostic prediction and subtyping of ESCC using lncRNA expression profiles. We also demonstrated a significant up-regulation of AC007128.1 in ESCC tumor tissues and cell lines and found its association with poor survival in ESCC patients. The mechanistic analysis demonstrated that AC007128.1 might promote cancer cell EMT by aberrantly activating the MAPK signaling pathway. These findings provided the possibility that overexpressed AC007128.1 was an important molecule participating in the aberrant activation of the MAPK signaling pathway and could serve as a therapeutic target for ESCC.

## Data Availability Statement

The data generated in the manuscript can be found in GEO using accession GSE167345.

## Author Contributions

SZ and JL designed the experiments. SZ performed the experiments, drafted the figures, and wrote the manuscript. HG and YT helped to perform the experiments. PL and YW provided direction in the experimental design. LD and CW revised the manuscript. All authors contributed to the article and approved the submitted version.

## Conflict of Interest

The authors declare that the research was conducted in the absence of any commercial or financial relationships that could be construed as a potential conflict of interest.

## Supplementary Material

Supplementary Figure 1 **(A–E)** The correlation between age, gender, alcohol, BE, and T stage and patients’ prognosis by KM analysis (log-rank test) (all *P* > 0.05).

Supplementary Figure 2 **(A–J)** The association between 10 out 11 prognostic lncRNAs and patients’ prognosis by KM analysis (log-rank test) (all *P* < 0.05). **(K)** LncRNA analysis of cp-score of patients with T1/2 and T3/4 (*P* = 0.18).

Supplementary Figure 3 **(A–E)** The dendrogram of different HC methods for ESCC patients from TCGA, among which the ward showed the best clustering. **(F)** The silhouette plot showed the optimal number of clusters (*k* = 2). **(G,H)** Silhouette analysis of the ward method **(G)** was slightly inferior to the *k*-means **(H)**. **(I)** Venn diagram showed the intersections of the three lncRNAs from diagnosis, prognosis, and subtyping sections. **(J,K)** OS for each of the lncRNA pattern in each subtype in both the training cohort (TCGA) and validation cohort (GEO) (log-rank test, *P* > 0.05).

Supplementary Figure 4 **(A,B)** Level of AC007128.1 in the nuclear and cytoplasmic fractions of KYSE150 and EC109 cells. **(C,D,H,I)** Growth curves of ESCC cells. Numbers of cells were determined at 24 h intervals after transfection with siAC007128.1-2 or pcDNA-AC007128.1 using the CCK-8 assay. **(E)** qPCR analysis of AC007128.1 expression in si-AC007128.1-2 treated TE-1 cells. **(F,G)** Representative micrographs of the transwell assay showing the migration and invasiveness of AC007128.1-depleted TE-1 cells. Data represent mean ± SEM from three independent experiments. ***P* < 0.01 and ****P* < 0.001 by Student’s *t*-test as compared with the corresponding control.
