# Supplementary figures and images for: lncRNA Profiles Enable Prognosis Prediction and Subtyping for Esophageal Squamous Cell Carcinoma
Source: Front Cell Dev Biol. 2021 May 28;9:656554. doi: 10.3389/fcell.2021.656554 (PMC8196240; doi:10.3389/fcell.2021.656554)

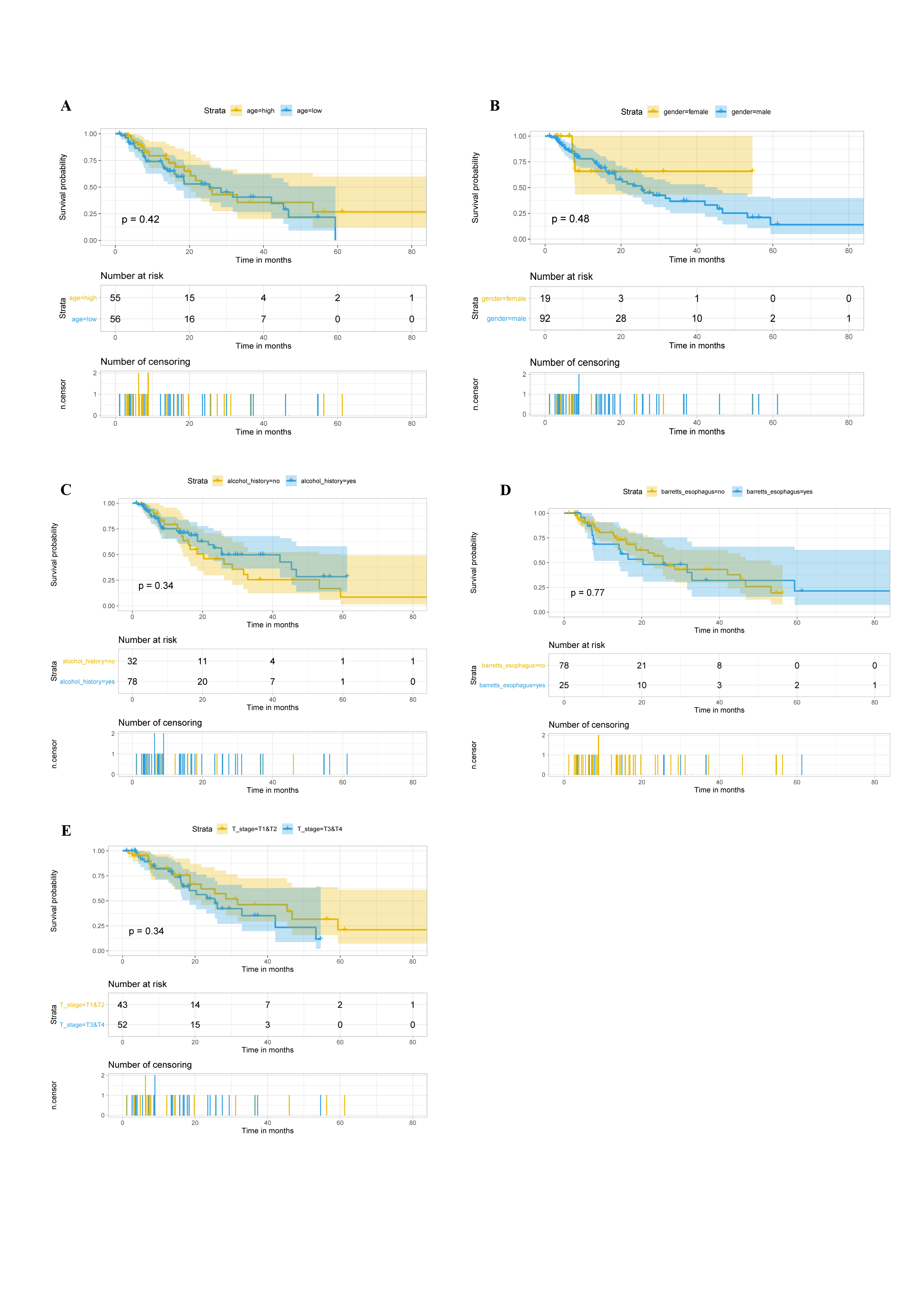

Supplement: Supplementary Figure 1 — (A–E) The correlation between age, gender, alcohol, BE, and T stage and patients’ prognosis by KM analysis (log-rank test) (all P > 0.05). [file Image_1.TIF]

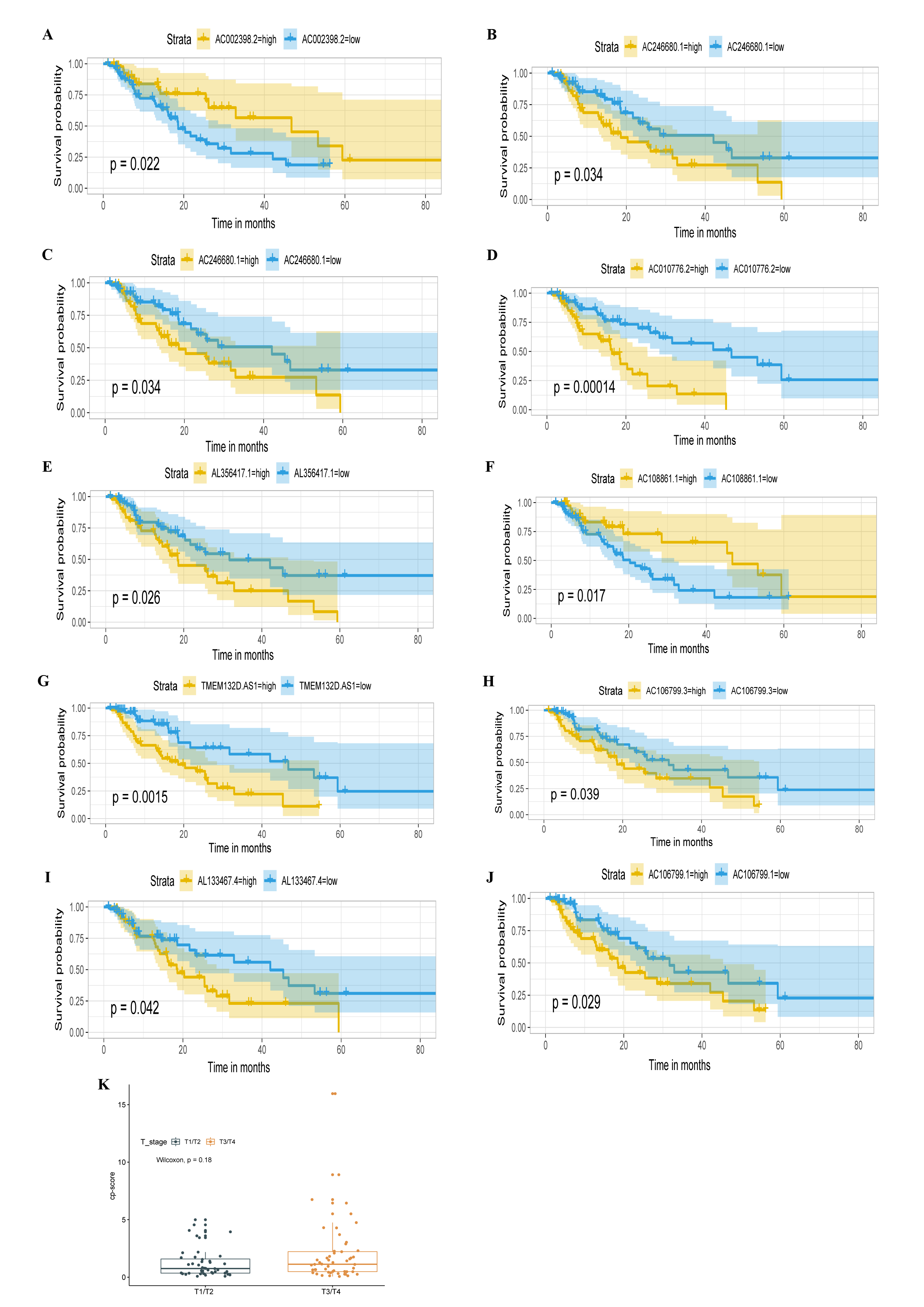

Supplement: Supplementary Figure 2 — (A–J) The association between 10 out 11 prognostic lncRNAs and patients’ prognosis by KM analysis (log-rank test) (all P < 0.05). (K) LncRNA analysis of cp-score of patients with T1/2 and T3/4 (P = 0.18). [file Image_2.TIF]

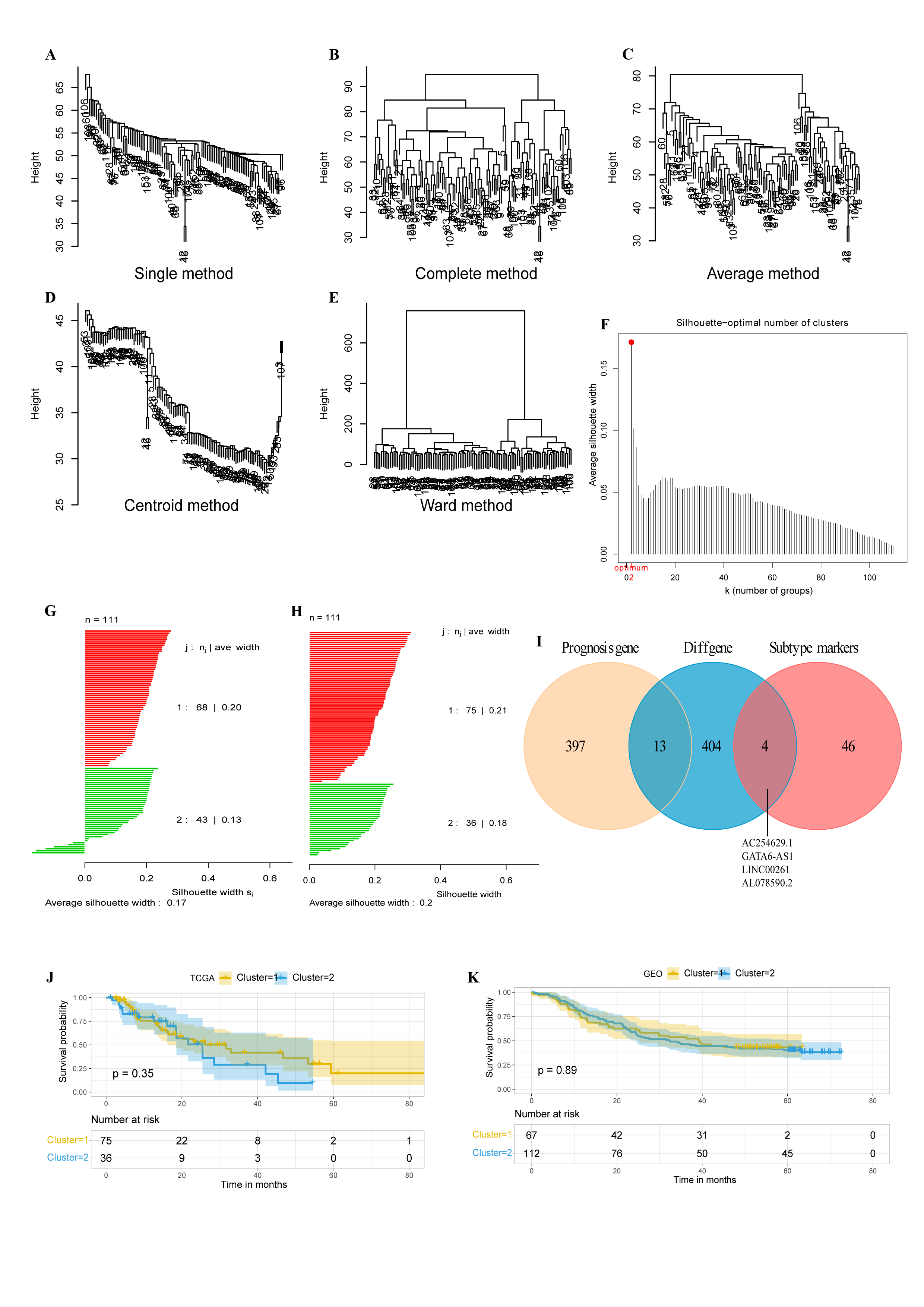

Supplement: Supplementary Figure 3 — (A–E) The dendrogram of different HC methods for ESCC patients from TCGA, among which the ward showed the best clustering. (F) The silhouette plot showed the optimal number of clusters (k = 2). (G,H) Silhouette analysis of the ward method (G) was slightly inferior to the k-means (H). (I) Venn diagram showed the intersections of the three lncRNAs from diagnosis, prognosis, and subtyping sections. (J,K) OS for each of the lncRNA pattern in each subtype in both the training cohort (TCGA) and validation cohort (GEO) (log-rank test, P > 0.05). [file Image_3.TIF]

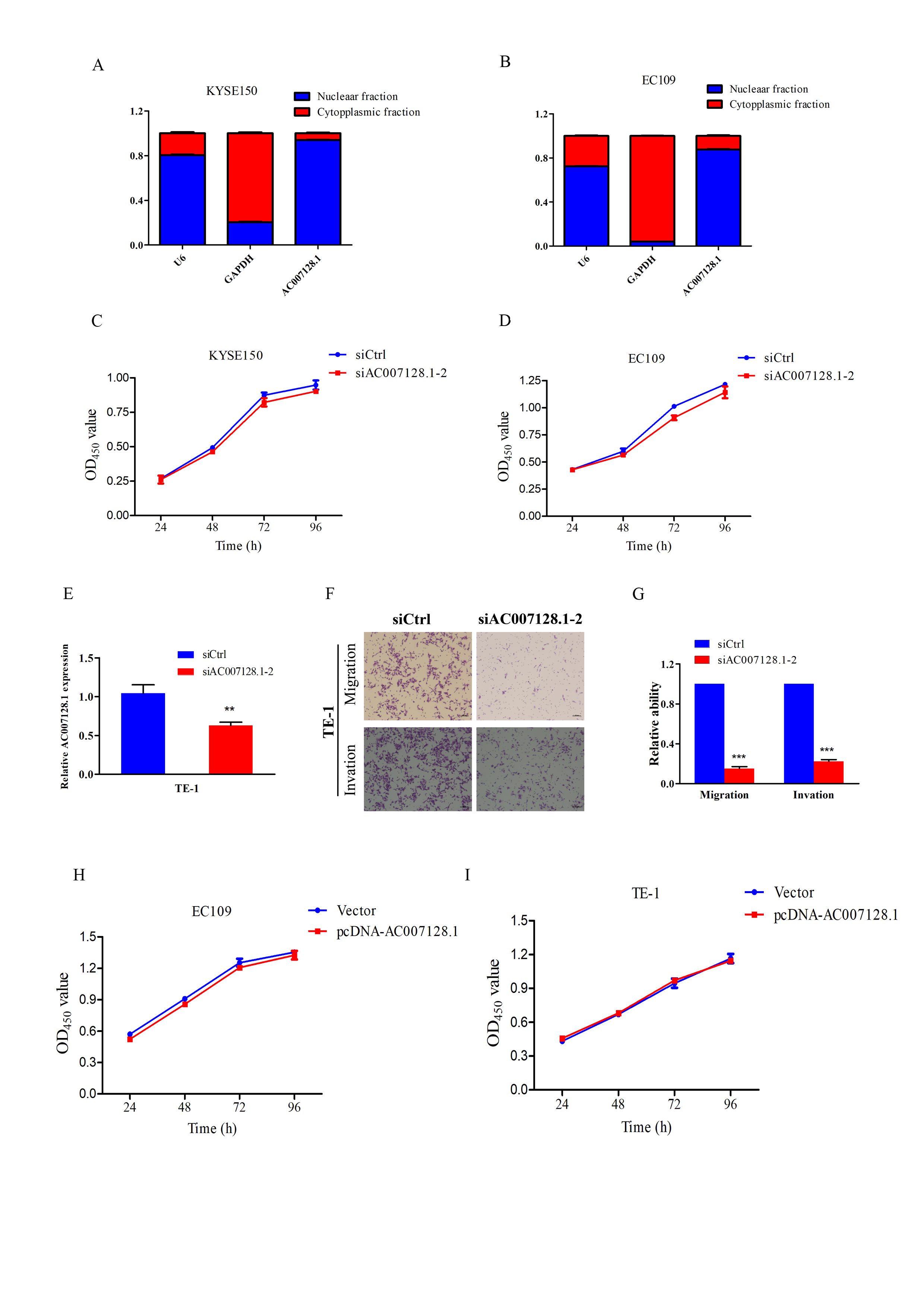

Supplement: Supplementary Figure 4 — (A,B) Level of AC007128.1 in the nuclear and cytoplasmic fractions of KYSE150 and EC109 cells. (C,D,H,I) Growth curves of ESCC cells. Numbers of cells were determined at 24 h intervals after transfection with siAC007128.1-2 or pcDNA-AC007128.1 using the CCK-8 assay. (E) qPCR analysis of AC007128.1 expression in si-AC007128.1-2 treated TE-1 cells. (F,G) Representative micrographs of the transwell assay showing the migration and invasiveness of AC007128.1-depleted TE-1 cells. Data represent mean ± SEM from three independent experiments. **P < 0.01 and ***P < 0.001 by Student’s t-test as compared with the corresponding control. [file Image_4.TIF]
